# The physiological roles of Sirt1 in skeletal muscle

**DOI:** 10.18632/aging.100312

**Published:** 2011-03-28

**Authors:** Patricia S. Pardo, Aladin M. Boriek

**Affiliations:** Division of Pulmonary, Critical Care and Sleep, Department of Medicine, Baylor College of Medicine, One Baylor Plaza 825E, Houston TX 77030

**Keywords:** aging, sirtuins, SIRT1, skeletal muscle, differentiation, nutrients

## Abstract

Skeletal muscle aging is associated with increased inflammation and oxidative stress, a decrease in the ability to rebuild muscle after injury and in response to exercise. In this perspective, we discuss the mechanisms regulating Sirt1 activity and expression in skeletal muscles, emphasizing their implications in muscle physiology and the impairment of muscle function with age.

## INTRODUCTION

SIRT1 (silent information regulator 1) is the mammalian homologue of the yeast Sir2, which is implicated in lifespan extension in model organisms, such as yeast, worms and flies [1-3]. It belongs to the family of sirtuins, the class III protein deacetylases that use NAD+ as a cofactor in such a way their activity is modulated by NAD+/NADH ratios that change with respiratory activity. The relevance of Sirt1 on normal physiology, development and aging is the result of its effect on chromatin remodeling by histone deacetylation and the modulation of a high number of non-histone substrates involved in transcriptional regulation [reviewed in 4]. Sirt1 regulates the activity of a variety of transcription factors and co-regulators with function in cell metabolism, growth, differentiation, survival, apoptosis, inflammation or stress response. In spite of the increasing knowledge about the protective role of Sirt1 on age-associated pathologies that include neurodegenerative and cardiovascular disease, metabolic disorders and cancer [reviewed in 5], the role of Sirt1 in muscle aging is poorly understood.

### Sirt1 role in muscle differentiation

**Figure 1.**
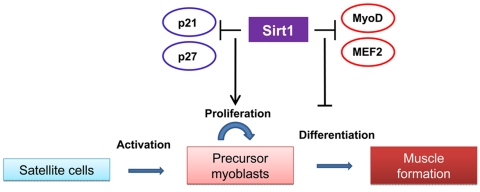
The role of Sirt1 in satellite cells proliferation and maturation The scheme summarizes the effects of increased Sirt1 expression observed in satellite cells and myoblasts on proliferation and myogenesis. In activated satellite cells. Sirt1 inhibits the expression of cell cyle inhibitors and promotes its proliferation. The negative role played by Sirt1 on the myogenic program by direct or indirect interactions with the myogenic factors MyoD and MEF2 suggest a further inhibition of muscle formation.

Sirt1 appears to play critical roles in muscle differentiation and metabolism. Several reports performed in skeletal muscle cells emphasize a negative effect of Sirt1 activation on myogenesis. Fulco et al., [6] found that Sirt1 expression and the NAD+/NADH ratio fall during muscle differentiation. C_2_C_12_ cells expressing exogenous Sirt1 fail to fully differentiate into myotubes whereas reducing Sirt1 levels by RNA interference results in differentiation of C_2_C_12_ and primary human skeletal myoblasts. The molecular mechanism responsible for the inhibition of myogenesis is attributed to the specific recruitment of Sirt1 to muscle regulatory regions on chromatin through its interaction with a MyoD/PCAF complex and the histone acetyltransferase GCN5, which controls the activity of the myogenic factor MEF2. These associations promote the deacetylation of histones on muscle specific enhancers and repression of the myogenic gene program. Further studies from the same authors [7] identified restricted glucose availability as one of the signals that inhibits muscle differentiation in a Sirt1 dependent manner by altering NAD+/NADH ratios. Such effect is mediated by activation of AMPK, a sensor of cellular energy levels, which results in enhanced expression of Nicotinamide monophorybosiltransferase, Nampt, the rate-limiting enzyme in the biosynthetic pathway that synthesize NAD+ from nicotinamide. Higher Nampt levels impact Sirt1 activity by increasing the availability of the Sirt1 substrate, NAD+, and by reducing the levels of the Sirt1 inhibitor, nicotinamide. Whereas these findings point out a negative role of Sirt1 in muscle formation, studies performed in muscle cell precursors, suggest that enhanced Sirt1 expression promotes their proliferation by inhibiting the expression of the cell cycle inhibitors p21^Waf/Cip1^ and p27^Kip1^ [8]. These authors identified low oxygen growth conditions as a factor controlling Sirt1 expression in muscle satellite cells. In this way, Sirt1 seems to be at the core of a mechanism controlling the balance between muscle cells precursors proliferation and differentiation in response to environmental cues (Figure 1). The understanding of how this balance is affected by aging should provide information about possible interventions to prevent the deficit in muscle hypertrophy in response to exercise and muscle repair after injury observed in aged animals and humans.

### Sirt1 role in muscle adaptation to nutrient availability and exercise

The roles played by Sirt1 in muscle metabolism are mainly attributed to its ability to deacetylate and activate the PPAR gamma coactivator 1, PGC-1, a transcription co-regulator of PPARs and other nuclear receptors. PGC-1 is induced by cold and beta-adrenergic agonists in skeletal muscle and brown fat [9] PGC-1 orchestrates the genetic program that allows skeletal muscle cells adaptation to meet the energetic demands driven by environmental stress, nutrient availability or increased muscle activity. Ectopic expression of PGC-1 in myotubes increases the expression of genes of the respiratory chain (e.g. cyto-chrome c and the cytochrome oxidase subunits II and IV) and promotes mitochondrial biogenesis by modulating the activity of the nuclear respiratory factor NRF1 and its target, mtfA, a nuclear factor that translocates to the mitochondria and activates mitochondrial DNA replication [10]. An important aspect of muscle plasticity is the ability to adapt to glucose deprivation by switching from glucose to lipid oxidation. PGC-1 is responsible for switching mitochondrial metabolism from glucose to fatty acid oxidation under conditions of starvation [11].

PGC-1 activity can be regulated by acetylation/deacetylation. GCN5 acetylates PGC-1 in liver and muscle, with a repressive effect on its activity [12,11] whereas Sirt1 deacetylates PGC-1 *in vitro* [13] and is required for fasting induced deacetylation of PGC-1 in skeletal muscle cells [11]. Studies performed in Sirt1 knocked down C_2_C_12_ myotubes and primary myoblasts evidenced its participation in PGC-1 mediated induction of genes that are involved in mitochondrial and fatty acid utilization metabolism in response to glucose deprivation [11]. The involvement of Sirt1 in mitochondrial biogenesis and oxidative metabolism *in vivo* is suggested by studies performed on mice fed with resveratrol, a potent activator of Sirt1. Resveratrol fed animals showed increased mitochondrial function evidenced as higher oxidative capacity, more resistance to muscle fatigue and increased muscle strength. Those physiological changes correlated with enhanced expression of genes involved in motor function and oxidative metabolism [14]. Recent findings demonstrated that AMPK, whose activity responds to changes in ATP/AMP ratios, is an upstream regulator of Sirt1 activity in muscles in response to exercise and fasting by controlling the enzymes of the NAD salvage pathway. Inhibition of AMPK activity prevents the deacetylation of PGC-1 by Sirt1 in skeletal muscle cells in response to glucose deprivation and downstream effects on respiratory activity and switch to fatty acid oxidation metabolism. Analysis of AMPKγ deficient mice evidenced impaired PGC-1 deacetylation in correlation with a failure to adapt muscle metabolism in response to fasting or exercise [15,16].

Consistently with its role in oxidative metabolism, PGC-1 and Sirt1 expression are higher in type I muscle oxidative fibers compared to type II glycolitic fibers. Transgenic mice developed to express PGC-1 under the muscle creatine kinase (MCK) promoter, highly expressed in fast twitch fibers, showed higher slow twitch fiber content, activation of oxidative metabolism and greater resistance to fatigue which are the hallmark of the slow twitch type I fibers [17]. In turn, skeletal muscle specific PGC-1 knock out mice showed a switch to fiber type IIb and IIx fibers accompanied by muscle damage and inflammation after endurance exercise [18]. The hypoxia-inducible factor 2 α (HIF2α) was recently identified as a PGC-1 target responsible for the switch to slow oxidative fibers. The involvement of Sirt1 in fiber type determination is suggested by its role in the modulation of the PGC-1 dependent transcriptional regulation of HIF2α [19]. A recent report by Amat et al. [20] provided experimental evidence that suggests that Sirt1 controls PGC-1 expression. This fact is suggested by lower PGC-1 RNA levels in muscles from Sirt1 deficient mice. The authors identified a molecular mechanism that requires the formation of a ternary complex formed by MyoD, Sirt1 and PGC-1 on a region of the PGC-1 promoter containing E2 boxes that results in an increased MyoD driven PGC-1 transcription and a positive regulatory loop of PGC-1 on its own transcription. Observations made in animals subjected to acute endurance exercise showed that the increase in Sirt1 protein expression occurs after two hours and largely precedes the increase in PGC-1 protein levels [21]. This phenomenon is consistent with the role of Sirt1 in the transcriptional regulation of PGC-1. These data suggest that Sirt1 is playing physiological roles independently of PGC-1. Recent findings from our group indicate that a fast induction of Sirt1 expression occurs in myotubes in response to mechanical stretch [22]. The regulation of Sirt1 expression by stretch occurs at the transcriptional level and requires the early growth factor 1, Egr1, whose expression is rapidly induced by mechanical stimulation. The stretch-dependent induction of Sirt1 by Egr1 is required to remove the excess of reactive oxygen species, ROS, generated by the mechanical stimulus. Sirt1 interaction with FoxO4 and expression and activity of Sod2 were identified as downstream targets of Sirt1. All these events occur in a short period and ROS detoxification occurs between 2 and 8 hours after stretch preventing a long exposure to ROS levels that might result in cell damage. Thus, ROS scavenging might be a consequence of the effects mediated by the fast induction of Sirt1 in response to exercise.

### Regulation of Sirt1 expression in skeletal muscle

The roles played by Sirt1 in muscle differentiation and adaptation to environmental cues have been mostly explained by changes in Sirt1 activity modulated by the availability of its cofactor NAD+ under the control of AMPK, thus sensing the redox and energy state of the cell [23]. Changes in Sirt1 expression occurs in skeletal muscles after fasting and endurance exercise suggesting coordination between the regulation of Sirt1 expression and Sirt1 activity. A study of the regulation of Sirt1 in liver and skeletal muscles in response to fasting revealed the involvement of FoxO3a and p53 in the transcriptional regulation of Sirt1. Under nutrient deprivation, FoxO3a shuttles from the cytoplasm to the nucleus and binds to p53 on two p53 responsive elements on the Sirt1 promoter [24].

Though there is evidence of decreased Sirt1 expression during myogenesis and increased Sirt1 expression by endurance exercise, the mechanisms controlling Sirt1 expression during these processes are completely unknown.

Our recent findings provide the first evidence of a mechanism controlling Sirt1 transcription by mechanical stimuli [22]. Sirt1 mRNA and protein levels increase by mechanical stretch in C_2_C_12_ myotubes and diaphragm muscles We previously found that FoxO3a was downregulated by stretch in diaphragms [25] and released from the Sirt1 promoter in diaphragms subjected to stretch (unpublished data). These data suggested that a distinct mechanism from the transcriptional activation of Sirt1 by nutritional stress, under the control of FoxO3a, was involved in the regulation of Sirt1 by stretch. In turn, Sirt1 induction by stretch correlated with Egr1 binding to a region of the Sirt1 promoter containing two Egr1 elements proximal to the p53 sites. In C_2_C_12_ myotubes, we proved the absolute requirement of Egr1 expression and the presence of one of the Egr1 elements for the transcriptional activation of the Sirt1 gene by stretch. Egr1 knock-down myotubes failed to induce Sirt1 in response to stretch and its consequent antioxidative response.

It has been previously established that electrical stimulation of the sciatic nerve, which induces intermittent exercise of the gastronemous elevates Egr1 mRNA levels within 45 minutes [26]. These results are consistent with the hypothesis that Egr1 is a possible candidate responsible for the increase in Sirt1 expression that occurs two hours after a bout of endurance exercise. Whole animal studies exploring the effect of acute exercise on Egr1 expression in muscles and Egr1 requirement for increased Sirt1 expression will highlight a relevant aspect of muscle adaptation to high levels of motor activity.

**Figure 2.**
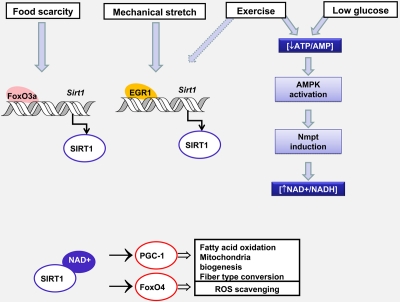
Changes in Sirt1 expression and activity controls muscle adaptation in response to environmental stress Nutrient availability and mechanical stretch promote Sirt 1 transcription by two different mechanisms involving FoxO3a or Egr1 recruitment to the Sirt1 promoter. Glucose restriction and exercise diminish the ATP/AMP ratio, activate AMPK which leads to an increase in NAD availability by inducing Nmpt expression. The consequent increase in Sirt1 content and/or activity modulates the transcriptional activity of FoxO4 and PGC-1. Whereas PGC-1 promotes oxidative metabolism and the conversion from glycolitic to oxidative fibers, FoxO4 triggers a response that prevents oxidative damage. Dashed arrows indicate hypothetical mechanisms.

In Figure 2, we summarize the mechanisms controlling Sirt1 activity and expression in response to nutritional stress, exercise and mechanical stretch and the physiological consequences of Sirt1 activation in muscle adaptation to environmental cues.

### Sirt1 role in the aging muscle

One of the hallmarks of skeletal muscle aging is an impairment of their regenerative potential. Muscle regeneration and growth in response to hypertrophic stimuli are driven by satellite cells, the myogenic progenitors that reside beneath the basal lamina of adult skeletal muscle, close to the myofiber surface. Satellite cells are quiescent in normal adult muscle and can be activated by muscle damage or other type of stress.

Activation is accompanied by a satellite cell self-renewal to maintain the reserve pool and production of satellite cell-derived myoblasts. This leads to the expression of MyoD and proliferation, later myogenin expression that marks the commitment to differentiation [27,28]. A reduction in satellite cells reservoir occurs with age that has been attributed to a reduced self-renewal capacity [29]. An involvement of Sirt1 in satellite cell proliferation has been reported, however these studies have been performed in MyoD expressing cells, suggesting they represent a population of activated satellite cells. Satellite cells from aged animals also have a reduced ability to differentiate [30] and increased nuclear content of Sirt1 [31]. These observat- ions suggest that though Sirt1 might play a positive role in the proliferation of activated satellite cells, higher levels of nuclear Sirt1 in the activated muscle progenitor cells of old animals may inhibit MyoD activity and diminish their myogenic potential. Considering that myoblasts precursors have adipogenic potential, this effect may be responsible for increased adiposity of aging muscles [32]. The understanding of the mechanisms controlling Sirt1 localization and expression in the myoblastic lineage will provide information that could potentially contribute to therapeutic interventions to counteract the negative age-associated changes in muscle regeneration.

**Figure 3.**
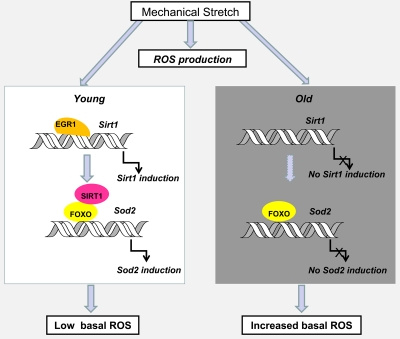
Hypothetical model of the effect of age on Sirt1 induction by stretch and its impact on age-associated oxidative stress in diaphragm muscles Egr1-dependent Sirt1 induction by stretch plays a role in recovering basal ROS levels through the activation of FOXO and increased Sod 2 expression. In the absence of alternative mechanisms of ROS detoxification, the loss of mechanoregulation of Egr1 binding to the Sirt1 promoter and Sirt1 expression in old compared to young diaphragm muscles, may explain the cumulative oxidative damage observed with age. Dashed arrows indicate hypothetical mechanisms.

Aging is also associated with a reduced muscle strength and functionality that are the consequences of mitochondrial dysfunction and cumulative mtDNA mutations, dysregulation of redox status, increase in markers of oxidative stress and inflammation [33-36]. Physical activity in older adults has been proven to partially counteract these effects [37,38]. However, the adaptation of muscles to resistance exercise that leads to improved muscular strength and fiber hypertrophy is decremented in older animals and humans [39,40]. Elevated oxidative stress has been proposed as a factor responsible for the attenuated adaptation to resistance exercise [41,42]. Our recent paper has clearly demonstrated that skeletal muscle cells growing in culture possess the ability to recover from the stretch-induced ROS production by activating Sirt1 expression which triggers an anti-oxidative response. Our unpublished data suggest that Sirt1 induction and increased Egr1 binding to the Sirt1 promoter is observed in stretched diaphragms of young mice but not in the muscles of old animals (Figure 3). The lack of mechanoresponsiveness of Sirt1 expression in old diaphragms may give account for an impaired ability to counteract the stretch-associated increase in ROS production leading to a sustained exposure to oxidative stress in this constantly active muscle.

The mechanisms controlling Egr1 expression in skeletal muscles are barely known. Studies in human muscle cells in culture suggests Egr1 induction occurs during muscle spindle formation by neuregulin, a heparin sulfate proteoglycan secreted by motor and sensor neurons in the neuromuscular junction [43]. A better understanding of the mechanism controlling Egr1 expression by mechanical stretch will allow defining proper interventions to prevent the deleterious effects of oxidative stress on muscle function at old ages.

## CONCLUSIONS

A decrease in motor function and the loss of the ability to regenerate or built muscle after injury are the two most relevant features of skeletal muscles aging. The roles played by Sirt1 in these two aspects are somewhat contradictory. Young mice treated with the Sirt1 activators, resveratrol and SRT1720, showed increased motor function [14,44] and a positive effect on muscle performance of the senescence accelerated mice SAMP1 by resveratrol treatment has been recently proven [45]. However, studies perform *in vitro* indicates that Sirt1, though promotes proliferation of myoblasts precursors [8], delays further maturation into myotubes [6,7] suggesting an overall negative impact on muscle repair. In this context, to be able to promote Sirt1 expression at the right moment appears to be crucial to improve motor function without impeding muscle formation. Interestingly, the stretch-dependent induction of Sirt1 by Egr1 is a transient mechanism which is shut down as a result of Sirt1 increased expression [23]. Thus, Egr1 emerges as a proper target that provides a way to increase Sirt1 levels during a short period and benefits from its effects on muscle adaptation to stress.
